# Inotropic effects of retatrutide in isolated human atrial preparations

**DOI:** 10.1007/s00210-025-04421-3

**Published:** 2025-07-04

**Authors:** Joachim Neumann, Undine Ahlrep, Britt Hofmann, Ulrich Gergs

**Affiliations:** 1 Institute for Pharmacology and Toxicology, Medical Faculty, Martin Luther University Halle-Wittenberg, Magdeburger Straße 4, D-06097 Halle (Saale), Germany; 2 Department of Cardiac Surgery, Mid-German Heart Centre, University Hospital Halle, Ernst-Grube-Straße 40, D-06097 Halle (Saale), Germany

**Keywords:** GLP-1, Glucagon receptor, GIP, Retatrutide, Human atrium, Mouse atrium

## Abstract

Retatrutide (LY3437943) was developed as a drug to treat type 2 diabetes and obesity. Retatrutide, a not endogenously occurring peptide, stimulated the glucagon receptor (GCGR), the glucose-dependent insulinotropic polypeptide (GIP) receptor (GIPR), and the glucagon-like peptide-1 receptor (GLP-1R) in cell cultures; increased the activity of adenylyl cyclases (AC); and thus augmented the 3′,5′ cyclic adenosine monophosphate (cAMP) levels. We tested the hypothesis that retatrutide increased force of contraction (FOC) in human right atrial preparations (HAP) from adult patients. HAP were obtained during open heart surgery from patients who suffered from severe coronary heart disease. We noted that cumulatively applied retatrutide starting at 10 nM (up to 100 nM the highest concentration tested) elevated FOC in HAP in a concentration- and time-dependent manner. In the additional presence of the phosphodiesterase III inhibitor cilostamide (1 µM), retatrutide was more potent and more effective to increase FOC in HAP. Under these conditions, retatrutide shortened the time of muscle relaxation in HAP. These positive inotropic effects of glucagon were diminished by a GLP1-R antagonist, by a GIPR antagonist, and by a CGCR antagonist but not by propranolol, an antagonist at β-adrenoceptors. The effects of retatrutide on FOC were also reduced by 100 nM ryanodine, an inhibitor of the ryanodine receptor in the sarcoplasmic reticulum, by 1 µM carbachol, a M-cholinoceptor agonist, and by 1 µM (-)-N^6^-phenylisopropyladenosine, an A_1_-adenosine receptor agonist. Summarily, we suggest that retatrutide enlarged FOC in HAP via the cAMP system through its cognate receptors.

## Introduction

Retatrutide was developed to treat diabetes type 2 and obesity (Jastreboff et al. 2022, Coskun et al. 2018, Coskun et al. 2022). These therapeutic effects were confirmed in a recent clinical trial (Jastreboff et al. 2023). Retatrutide could activate GCGR, GIPR, and GLP1-R in transfected cells in cell culture to raise cAMP levels (Coskun et al. 2022). Retatrutide increased cAMP in these receptor-transfected cells with affinities of (in nanomolar concentrations): 5.8, 0.06, and 0.78 at GCGR, GIPR, and GLP1-R, respectively (Coskun et al. 2022). In mouse atrial preparations, these receptors are present, at least on the mRNA levels (Neumann et al. 2023a, Neumann et al. 2023b, Ussher et al. 2018). Whether these receptors (GCGR, GIPR, GLP-1R) are expressed as proteins in the mouse atrium is currently not clear because the specificity of commercial antibodies against these three receptors is doubtful and controversial (Neumann et al. 2023b).

In both monkeys and humans, retatrutide elevated the heart rate and decreased the blood pressure (Coskun et al. 2022, Jastreboff et al. 2023). This does not allow us to decide whether that retatrutide acts directly or indirectly on the human heart.

Hence, we started the present study to find out whether or not retatrutide can affect directly the mechanical function of the human heart, using HAP as our model system. In a different communication, we also studied the function of retatrutide in the mouse heart (abstract: Ahlrep et al. 2025). In the mouse atrial preparations, GLP1-receptor agonists do not increase FOC or beating rate (Neumann et al. 2024a). GIP increases FOC only in the presence but not in the absence of a phosphodiesterase inhibitor (Neumann et al. 2024b). GIP failed to increase the beating rate in isolated mouse atrial preparations (Neumann et al. 2024b). Glucagon failed to increase FOC but exerted a potent positive chronotropic effect in mouse left atrial preparations or mouse right atrial preparations, respectively (Neumann et al. 2023a, Neumann et al. 2023b, Neumann et al. 2024a). Glucagon alone was unable to increase FOC in isolated HAP (Aranda-Domene et al. 2023). However, in the presence of the phosphodiesterase III-inhibitor cilostamide, glucagon could increase FOC in HAP (Neumann et al. 2025). Even in the absence, but more potent and effective in the presence of cilostamide, GIP increases FOC in HAP (Neumann et al. 2024b). Finally, GLP-1R agonists increased FOC alone in the HAP (Wallner et al. 2015). In the presence of cilostamide, GLP-1R agonists were more potent and effective to raise FOC than in the absence of cilostamide (Neumann et al. 2024a). Hence, it was an open question whether or not a “triple” agonist like retatrutide would augment FOC in HAP. Should a positive inotropic effect of retatrutide exist, then the question would arise, which receptor(s) would mediate this FOC in HAP. A progress report of this study has already appeared in abstract form (Ahlrep et al. 2025).

Thence, we tested mainly the following hypotheses:

1. Retatrutide exerts a positive inotropic effect in HAP.
2. This positive inotropic effect is mediated by GLP-1R.
3. This positive inotropic effect is mediated by GCGR.
4. This positive inotropic effect is mediated by GIPR.

## Material and methods

### Contractile studies on human cardiac preparations

**Fig. 1:**
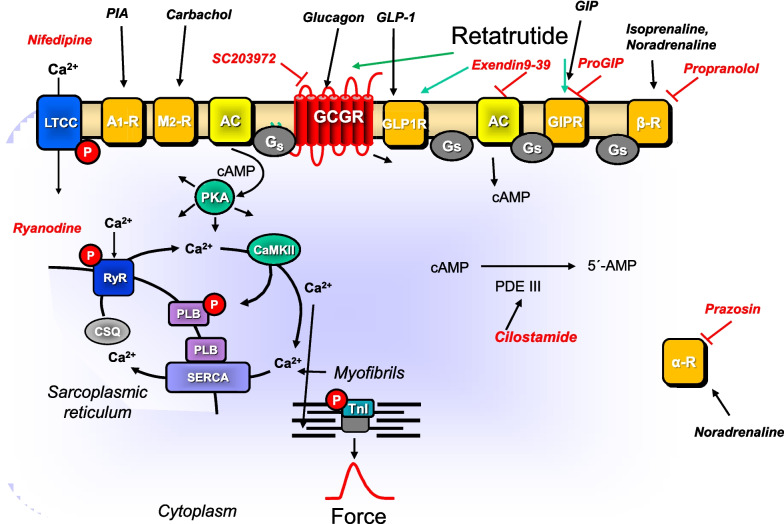
Signal transduction in HAP. Putative mechanism(s) of action of retatrutide in cardiomyocytes. Glucagon stimulates glucagon receptors (GCGR, Sutherland and Rall 1958; Glick et al. 1968, GCGR antagonized by SC 203972). Glucagon-like-protein-1 (GLP-1) stimulates GLP-1 receptors (GLP-1R, Holst 2007, antagonized by exendin9-39). Glucose-dependent insulinotropic polypeptide (GIP) stimulates the GIP receptors (Heimbürger et al. 2020, GIPR, antagonized by ProGIP). Then via stimulatory GTP-binding proteins (Gs), adenylyl cyclase (AC) catalyzes the formation of cAMP. This cAMP activates a cAMP-dependent protein kinase (PKA). This PKA activates by phosphorylation (P) cardiac regulatory proteins. The cAMP is degraded to 5′-AMP in the human heart mainly by phosphodiesterase III that can be inhibited by cilostamide. PKA can phosphorylate phospholamban (PLB). SERCA pumps Ca^2+^ from the cytosol into the sarcoplasmic reticulum. Ca^2+^ binds to calsequestrin (CSQ). RyR indicates the ryanodine receptor (inhibited by ryanodine) which releases Ca^2^ from the sarcoplasmic reticulum. LTCC means the L-type Ca^2+^ channel which is inhibited by nifedipine. Myofibrils, in which PKA can phosphorylate the inhibitory subunit of troponin (TnI), are responsible for the Ca^2+^ dependent generation of force which is symbolized here by a single muscle contraction over time. PIA stimulates A_1_-adenosine receptors. Carbachol stimulates the M_2_-muscarinic receptor. The β-adrenoceptor is stimulated by isoprenaline or endogenous noradrenaline and inhibited by propranolol. The α-adrenoceptor is stimulated by endogenous noradrenaline and inhibited by prazosin

**Fig. 2:**
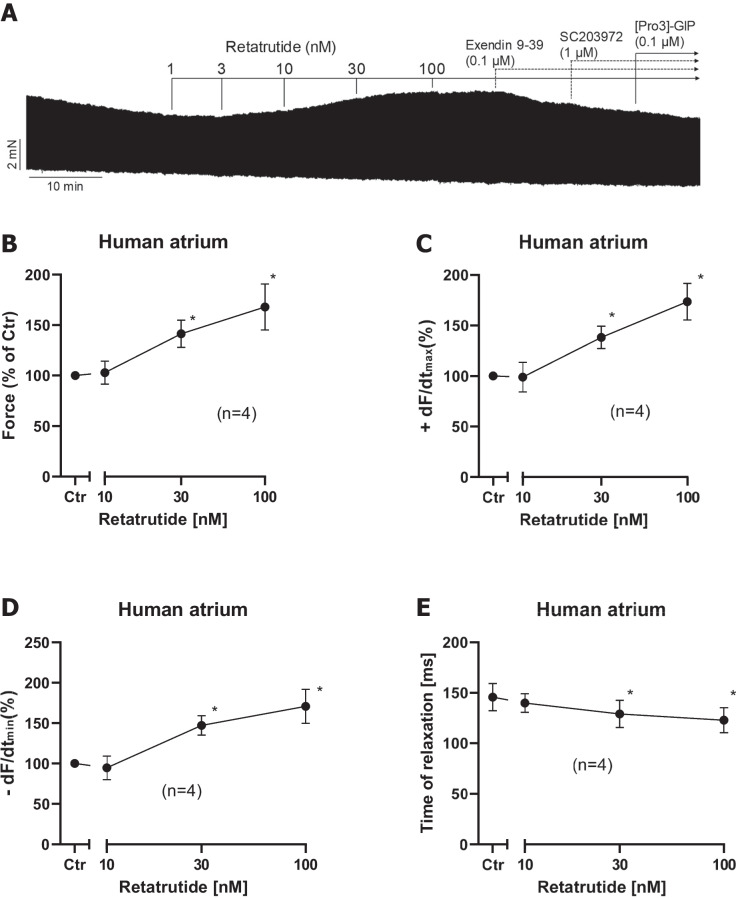
Retatrutide alone increases FOC in HAP. **A** Original recording of FOC in HAP. Addition of drugs is indicated by vertical lines. Ordinates give FOC in mN. Data from several experiments like in **A** were subsequently summarized. Ctr means force in % before addition of any retatrutide (**B**), rate of tension development (**C**), rate of tension relaxation (**D**), and time of relaxation (**E**) in milliseconds. Ordinates indicate force in percentage of pre-drug value in **B**, rate of tension development in percentage of pre-drug value in **C**, rate of relaxation in percentage of pre-drug value in **D**, and time of relaxation in milliseconds (ms) in **E**. Abscissae indicated negative decadic logarithm of the concentration of retatrutide. *p < 0.05 vs. Ctr (pre-drug value). Number in brackets give number of experiments

**Fig. 3:**
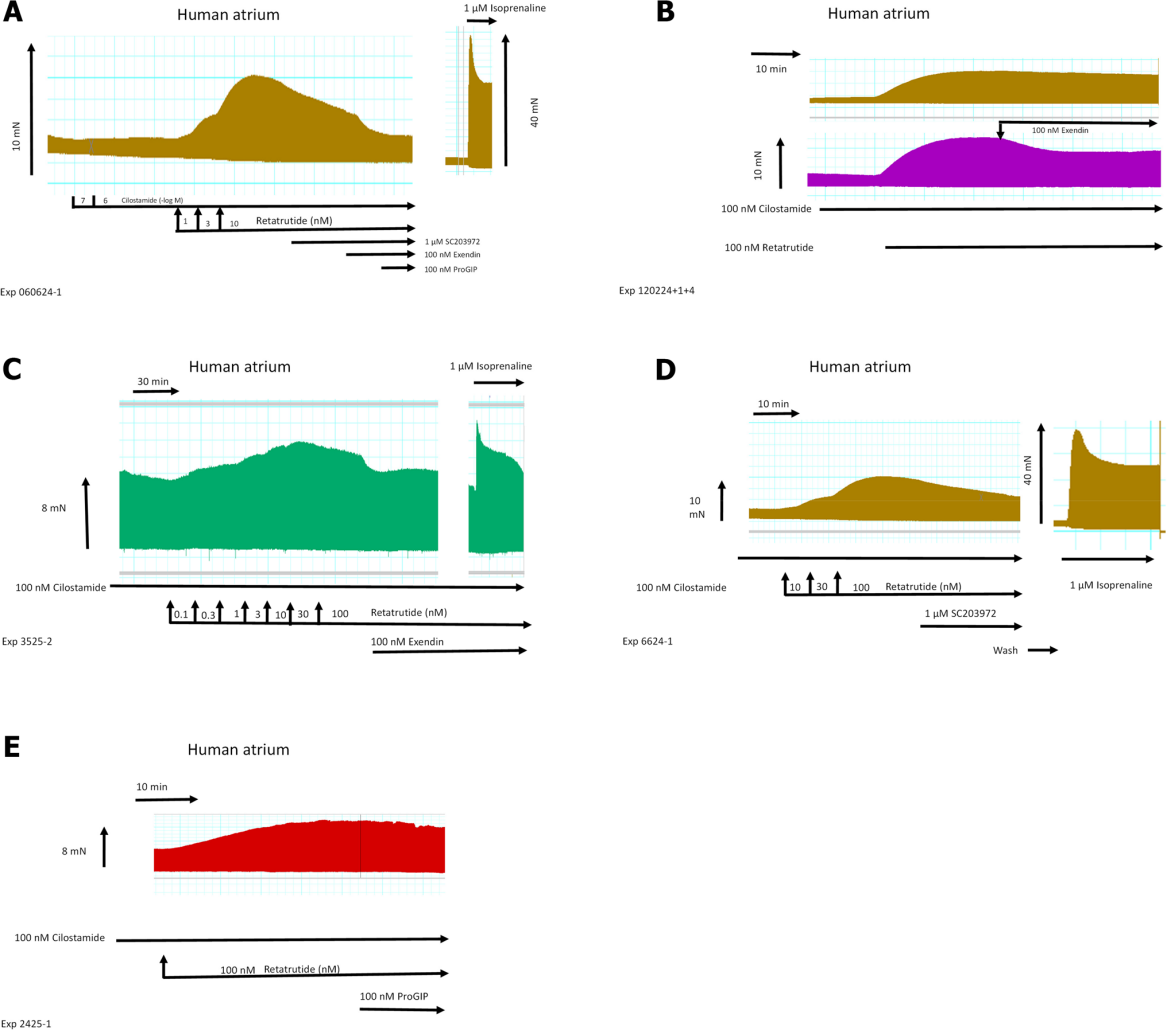

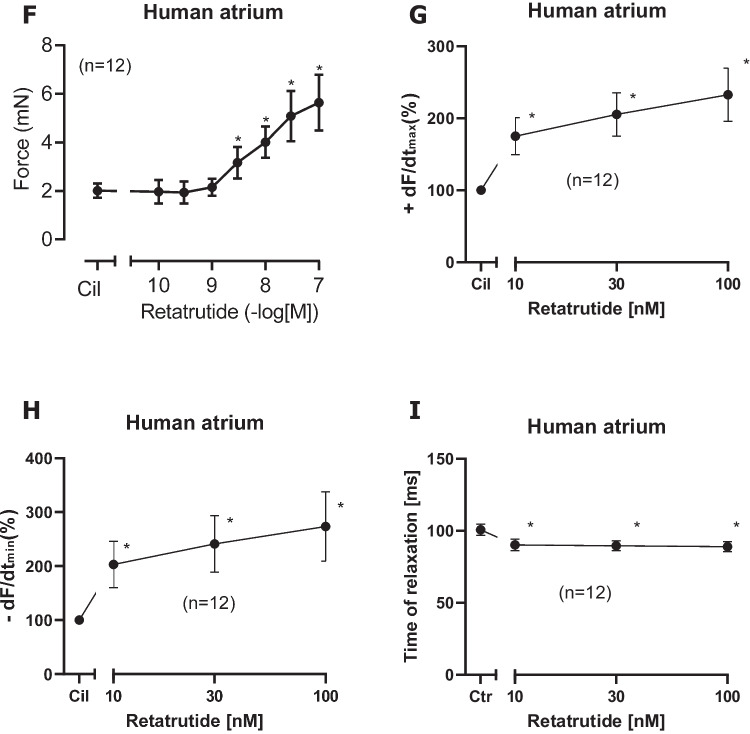
Retatrutide in the presence of cilostamide, a phosphodiesterase III inhibitor, increases FOC in HAP. Typical original recording (**A**): first, cilostamide was given; then, retatrutide was cumulatively applied. Finally, glucagon receptor antagonist SC203972, GLP-1R antagonist exendin 9–39, and the GIPR antagonist [Pro3]GIP were sequentially added to the organ bath. Please note that on the right hand side, the effect of isoprenaline was so strong that a different scale was required. Please also note that we first applied 100 nM cilostamide, and when we failed to detect any increase in force of contraction, we added 1 µM cilostamide to raise tension. In **B** (top), a time control for the positive inotropic retatrutide in the presence of cilostamide is presented. In **B** (bottom), the negative inotropic effect of exendin is displayed. In **C**, the negative inotropic effect of exendin after a concentration response curve to retatrutide in the continuous presence of cilostamide is shown. Please note that at the right hand side, the positive inotropic effect of isoprenaline after drug washout is depicted. In **D**, the negative inotropic effect of SC203972 after retatrutide in the presence of cilostamide is shown. On the right hand side, the effect of isoprenaline after drug wash out is seen. Please note that different scales are used for isoprenaline to indicate force in mN. Finally, in **E**, the negative inotropic effect of Pro(3)GIP (ProGIP) is shown after retatrutide and cilostamide. Ordinates in **A–E** give force of contraction in milli Newton (mN). Horizontal bar indicates time of the experiment in minutes (min). SmVertical arrows indicate time where the indicated drugs were added. Larger arrows on the left hand side, in the middle, or the right hand side indicate force of contraction. Data from the first part with retatrutide coming from several experiments like in **A** were subsequently summarized. Here, Ctr means force in milli Newton (mN) before addition of any retatrutide. Moreover, force of contraction in mN (**F**), rate of tension development in mN/seconds (mN/s, **G**), rate of tension relaxation (**H**), and time of relaxation (**I**) in milliseconds (ms) are plotted. Ordinates indicate the rate of tension development in percentage of pre-drug value in **G**, rate of relaxation in percentage of pre-drug value in **H**, and time of relaxation in milliseconds (ms) in **I**. Abscissae indicate nanomolar (nM) concentrations of retatrutide. *p < 0.05 vs. Cil (value of the contractile effect of cilostamide. Number in brackets give number of experiments

In brief, human atrial preparations obtained during the cardiac surgery at the sites where extracorporeal circulation needles were inserted into the right atrial appendage and were rapidly (within 30 min) transferred into the laboratory in flasks filled with a modified Tyrode’s solution. The contractile studies on HAP were performed using the same modified Tyrode’s solution. Muscle strips were stretched to optimal length that allowed maximal generation of FOC. The modified Tyrode’s solution contained in millimolar concentrations (mM): 119.8 NaCI, 5.4 KCI, 1.8 CaCl_2_, 1.05 MgCl_2_, 0.42 NaH_2_PO_4_, 22.6 NaHCO_3_, 0.05 Na_2_EDTA, 0.28 ascorbic acid, and 5.05 glucose. Ascorbic acid is used here as an antioxidant to maintain the activity of, for instance, isoprenaline. The solution was continuously gassed with 95% O_2_ and 5% CO_2_ and maintained at 37 °C and pH 7.4 in the organ baths. HAP were stimulated (60 beats per minute, bpm) electrically with platinum electrodes with rectangular impulses of direct currents from a Grass stimulator SD 9 (Quincy, Massachusetts, USA). Voltage was set around 10 V, just sufficient to initiate contractions. Electrical impulses had a length of 5 ms. The signals from the force transducer were fed into a bridge amplifier (from Hugo Sachs company, Freiburg, Germany) and digitized and stored on a commercial personal computer. The signals were quantified using a commercial software (Lab Chart 8 from ADInstruments bought through their distributor in Oxford, England). Our methods used for atrial contraction studies in human samples have been previously published and were not altered in this study (e.g., Gergs et al. 2017, 2019). The muscle strips were then mounted under isometric conditions with metal hooks at each end of the muscle in a glass organ bath. The HAP were obtained from 24 male patients and five female patients, aged 52–84 years. The patients suffered from severe coronary diseases (two and three vessel diseases). The cardiac drug therapy included acetylsalicylic acid, apixaban, furosemide, and metoprolol. Cardiac comorbidities included in addition to coronary heart disease also hypertension and atrial fibrillation (Fig. 1, 2). In some experiments, we applied receptor antagonists, an ion channel antagonist or a phosphodiesterase inhibitor to the organ baths as delineated in the figure legends and in Table 1. Indeed, we usually need only 100 nM cilostamide to elicit an increase in force. In rare case, we increase to 300 nM and even 1 µM cilostamide, if we fail to detect an increase in force with 1 µM cilostamide (see Fig. 3A). We always try to detect a small inotropic effect of cilostamide before we add other drugs that act via cAMP and are thus expected to be more effective in the presence of cilostamide. We used this procedure successfully in previous work to amplify effects on GLP-1R and GCGR (e.g., Neumann et al. 2025a, Neumann et al. 2025b).

**Table 1:** Receptors antagonists, an ion channel antagonist, or a phosphodiesterase inhibitor

Drug	Receptor/channel	Mechanism of action
Glucagon	GCGR	Agonist
GLP1	GLP-1R	Agonist
GIP	GIPR	Agonist
SC203972	GCGR	Antagonist
Pro(3)GIP (ProGIP)	GIPR	Antagonist
Exendin 9–39 (Exendin)	GLP-1R	Antagonist
Carbachol	M_2_-cholinoceptor	Agonist
PIA (R-PIA)	A_1_-adenosine receptor	Agonist
Prazosin	α_1_-adrenoceptor	Antagonist
Isoprenaline	β-adrenoceptor	Agonist
Propranolol	β-adrenoceptor	Antagonist
Nifedipine	L-type calcium channel	Inhibitor
Cilostamide	Phosphodiesterase III	Inhibitor
Ryanodine	Ryanodine receptor	Antagonist

Legend to Table 1, compare Fig. 1: *GCGR*, glucagon receptor; *GLP1R*, glucagon-like-protein-1 receptor; *GIPR*, glucose-dependent insulinotropic polypeptide receptor. Drugs are, in part, listed in the “Material and methods”

**Fig. 4:**
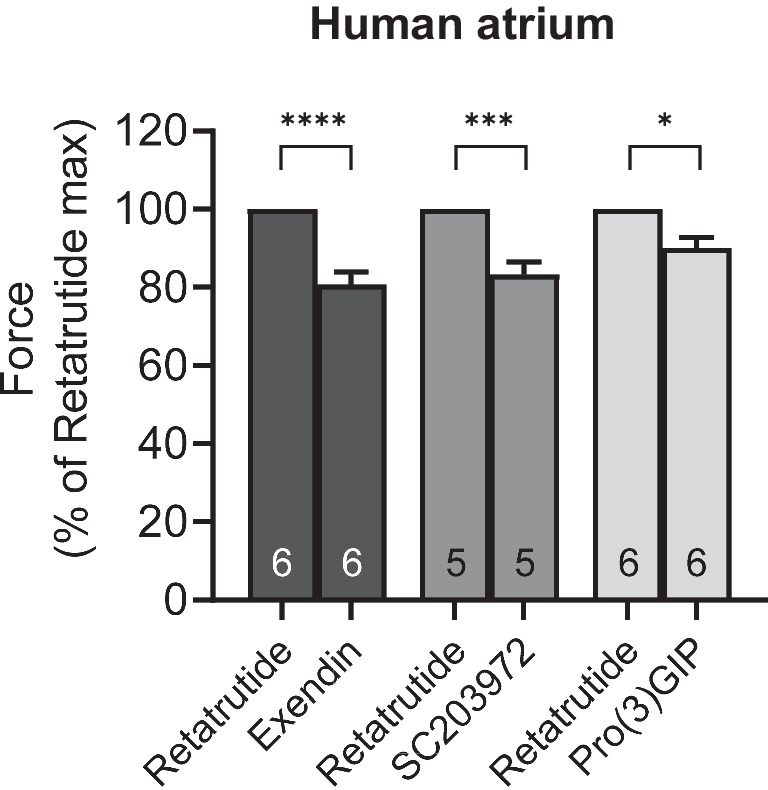
Antagonists reduce the positive inotropic effects of retatrutide in the presence of cilostamide in HAP. Effect of antagonists (exendin, SC203972, Pro(3)GIP) in the presence of cilostamide on retatrutide-stimulated FOC in HAP. Bars indicate FOC before the listed antagonists. HAP were treated in three different groups. All three groups received first cilostamide and retatrutide (see Fig. 3A) up to 100 nM: then, the first group received exendin (such as in Fig. 3B), the second group was treated with SC203972 (such as in Fig. 3D), and the third group was incubated with Pro(3)GIP (such as in Fig. 3E). In each muscle, the value at 100 nM retatrutide was set as 100%. Numbers in bars indicate the number of experiments. * indicate *p* < 0.05 versus pre-antagonist force generated with 100 nM retatrutide

**Fig. 5:**
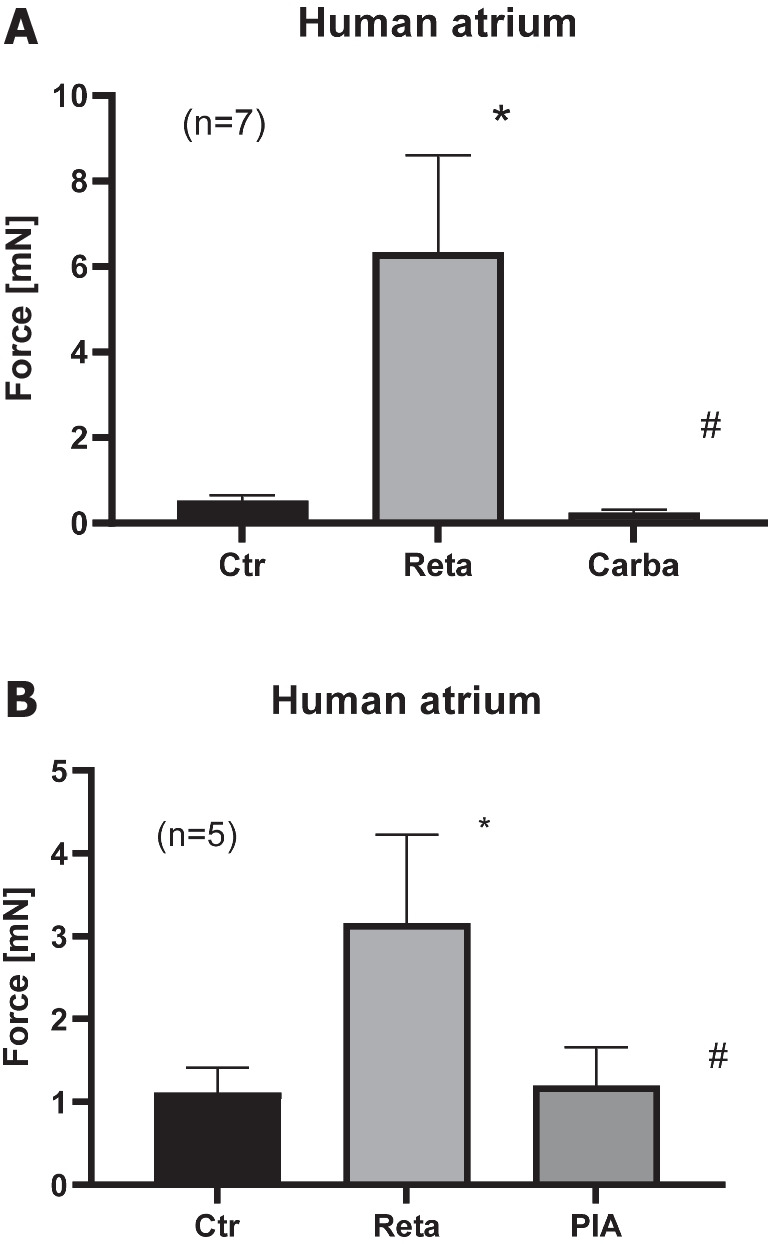
Carbachol and PIA reduced retatrutide stimulated force in the presence of retatrutide in HAP. **A** Effect of retatrutide (Reta) in the presence of cilostamide on FOC in milli Newton (vertical bars, mN) in electrically stimulated human right atrial muscle strips. Bars indicate FOC before retatrutide addition (Ctr), maximum FOC of 10 nM retatrutide (Reta), and maximum effect of 1 µM carbachol (Carba). Number in brackets indicates the number of experiments. * and # indicate *p* < 0.05 versus Ctr or Reta, respectively. **B** Effect of retatrutide (Reta) in the presence of cilostamide on FOC in milli Newton (vertical bars, mN) in electrically stimulated human right atrial muscle strips. Bars indicate FOC before retatrutide (Reta) addition (Ctr), maximum FOC of 10 nM retatrutide (Reta), and maximum effect of 1 µM PIA (PIA). Number in brackets indicates the number of experiments. * and # indicate *p* < 0.05 versus Ctr or Reta, respectively

**Fig. 6:**
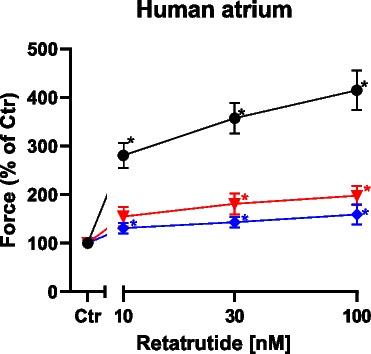
Nifedipine and ryanodine in the presence of cilostamide reduce the positive inotropic effect of retatrutide in HAP. The conditions were tested: Group A: first, cilostamide was given; then, retatrutide was cumulatively applied (black circles, *n* = 9). Group B: cilostamide was given, then 100 nM nifedipine, and then retatrutide was cumulatively applied (red triangles, *n* = 5). Group C: cilostamide was given, then 10 nM ryanodine, and then retatrutide was cumulatively applied (blue diamonds, *n* = 5). Ordinate gives force of contraction in percentage of control (Ctr: pre-drug values). Abscissa gives concentration of retatrutide in nanomolar (nM). Asterisks give significant differences (*p* < 0.05) against the appropriate color-coded value of Ctr. With two-way ANOVA, the curve of nifedipine (red triangles) was significantly different (*p* = 0.0008) against the black control curve (closed circles). With two-way ANOVA, the curve of ryanodine (blue diamonds) was significantly different (*p* = 0.0001) from the control curve (black closed circles)

Drug addition was cumulatively or non-cumulative as described in the figure legend. For Fig. 4, 5, we gave after cilostamide and retatrutide three different drugs, either an GCGR inhibitor or an inhibitor at GLP1-R or an inhibitor at GIPR, as can be seen in original recordings in Fig. 3. For the experiments in Fig. 6, we first gave cilostamide and then either ryanodine or nifedipine or as a control neither ryanodine nor nifedipine. Thereafter, increasing concentrations of retatrutide were cumulatively applied (Fig. 6).

### Data analysis

Data shown are mean ± standard error of the mean. Statistical significance was estimated using Student’s t-test or the analysis of variance followed by Bonferroni’s t-test as described in the legends. A *p*-value < 0.05 was considered to be significant.

### Drugs and materials

(-)Isoprenaline tartrate was from Sigma Aldrich (Taufkirchen, Germany) and dissolved in water. Pro(3)GIP came from Bachem (Bubendorf, Switzerland) and was dissolved in water. We used 100 nM exendin, 100 nM ProGIP, and 1 µM SC203972 based on our contraction data in HAP (Neumann et al. 2023a, 2024a). The concentrations of exendin9-39, SC203972, and Pro(3)GIP were based on beating rate in mouse atria and on binding data on recombinant receptors in the literature (Boknik et al. 2009, Neumann et al. 2003, Neumann et al. 2023a, Neumann et al. 2023b, Neumann et al. 2024a, Neumann et al. 2024b). Exendin9-39, SC203972, and Pro(3)GIP at the concentrations used failed to affect force of contraction (Neumann et al. 2023b, Neumann et al. 2024a, 2024b). These concentrations were higher than the affinity constants at the receptors of interest and therefore should have been adequate. The concentrations of carbachol, PIA, nifedipine, and ryanodine were based on our own work (Neumann et al. 2024a; Schwarz et al. 2023, 2024). All other chemicals were of the highest purity grade commercially available. Deionized water was used throughout the experiments to prepare the modified Tyrode’s solution. Stock solutions were prepared fresh daily.

## Results

In HAP, cumulatively applied retatrutide alone (in the absence of a PDE inhibitor) elevated FOC within 10 min of incubation starting at 10 nM and reaching plateau at 100 nM retatrutide. This can be seen in an original recording (Fig. 2A). Several such experiments are summarized in % of pre-drug value (Fig. 2B). Like isoprenaline, retatrutide raised the rate of tension development and the rate of tension relaxation (Fig. 2C and Fig. 2D). Like isoprenaline, retatrutide shortened time of relaxation (Fig. 2E). The effect of additionally applied 100 nM exendin 9–39 (a GLP-1R antagonist) or ProGIP (a GIPR antagonist) or a SC203972 (a GCGR antagonist) is also seen in (Fig. 2A). We did not try to quantify the effect of exendin, ProGIP, and SC203972 on the positive inotropic effect of retatrutide because we later noticed that retatrutide was more effective in the presence of the phosphodiesterase III-inhibitor cilostamide. We therefore studied these receptor antagonist only after cilostamide and retatrutide (original tracings in Fig. 3) and therein quantified any effects (Fig. 4).

Indeed, we hypothesized that when cAMP degradation was inhibited, retatrutide might be more potent and effective to raise FOC. This turned out to be true. When we gave low concentrations of cilostamide alone, a PDE III inhibitor, we and others routinely used in HAP (Berk et al. 2016, Neumann et al. 2025) to the organ bath, we elevated FOC. Then, we added retatrutide to raise FOC further. This is depicted in a typical original experiment in Fig. 3A. Retatrutide exerted in the presence of cilostamide a concentration- and time-dependent positive inotropic effect (Fig. 3A). Several experiments in milli Newton are depicted in Fig. 3F. Such experiments also depicted that retatrutide increased the rate of tension development and the rate of tension relaxation (Fig. 3G and Fig. 3H). Moreover, retatrutide reduced time of relaxation (Fig. 3I). The positive inotropic effect of retatrutide in the presence of cilostamide (Fig. 3A) could be reduced greatly by the antagonist exendin(9–39), Pro(3)GIP, and SC203972. This is seen in a typical original recording (Fig. 3A). We did not try to quantify these successive negative inotropic effects of receptor antagonists as one antagonist may have altered the effect of another. Three such groups were stimulated with cilostamide and retatrutide. Then, each group received first exendin(9–39) (Fig. 3B, Fig. 3C) or first SC203972 (Fig. 3D) or first Pro(3)GIP (Fig. 3E). Several such experiment are summarized in Fig. 4. Similarly, carbachol and PIA attenuated the positive inotropic effect of retatrutide (Fig. 5A, Fig. 5B).

Moreover, one could imagine that retatrutide like, e.g., amphetamine (Neumann et al. 2024a, b) might be at least in part an indirect sympathomimetic agent. Hence, we performed experiments where we first gave 10 µM propranolol and 10 µM prazosin. Then, we added 100 nM cilostamide and then 100 nM retatrutide. Under these conditions, retatrutide still about doubled FOC (increase by 289% after 100 nM compared to force before retatrutide, or from 2.01 ± 0.33 mN to 7.29 ± 1.05 mN, *n* = 8).

Moreover, the question arose whether the positive inotropic effect of retatrutide (in the presence of cilostamide) like those of isoprenaline was mediated in part by increasing the current through the L-type calcium cation channel. Therefore, we first applied cilostamide and retatrutide and then we added 100 nM nifedipine. Under these conditions, we noted that nifedipine reduced the positive inotropic effect of retatrutide to 66.3 ± 7.82% (from 5.67 ± 1.09 mN to 3.17 ± 0.63 mN, *n* = 5, *p* < 0.05). In addition, in a new set of experiments, we used an alternative protocol: we first gave cilostamide, then added 100 nM nifedipine, and then added retatrutide. For comparison, we used control times that were lacking nifedipine (see Fig. 6). Thus, nifedipine reduced the efficacy of retatrutide to increase force of contraction.

Finally, one may ask whether the release of calcium cations via ryanodine receptors from the sarcoplasmic reticulum contributed to the positive inotropic effect of retatrutide. Therefore, we first applied cilostamide and retatrutide. When the positive inotropic effect had reached plateau, we added 10 nM ryanodine. Under these conditions, we noted that ryanodine reduced the positive inotropic effect of retatrutide. More specifically, when we defined the maximum positive inotropic effect of retatrutide as 100%, then the maximum negative inotropic effect of ryanodine amounted to 31.1 ± 4.58% (from 5.25 ± 1.66 mN to 1.23 ± 0.36 mN, *n* = 7). In addition, we used an alternative protocol in new set of experiments where we studied (in the presence of cilostamide) the effect of retatrutide, ryanodine, and nifedipine or control on different muscle strips from the same patient head to head: we first gave cilostamide, then added 10 nM ryanodine, and then added retatrutide (Fig. 6). Thus, ryanodine reduced the efficacy of retatrutide to increase force of contraction.

## Discussion

Here, our main new finding is that retatrutide can exert a positive inotropic effect in HAP (Fig. 6). This could be of clinical relevance because retatrutide has entered at least ten clinical studies (at clinicaltrials.gov, at the day of this writing). Hence, the drug producer will try to get this drug approved. We assume competitive drugs with this “triple” mechanism are in the pipeline.

Retatrutide (8 mg) led to peak plasma concentrations of 874 ng/ml (182 nM) (Coskun et al. 2018). This means that the positive inotropic effect that we described in this report occurred at plasma concentrations that are found in patients. Hence, this supports our conclusion that our findings are of potential clinically relevant.

These positive inotropic effects are probably mediated by three receptors in HAP. These conclusions are supported by the observation that the positive inotropic effect of retatrutide was attenuated, in part, by a GIP receptor antagonist, in part by a glucagon receptor antagonist and in part by a GLP-1 receptor antagonist. In addition, we report on a partial contribution of the glucagon receptor and of the GIPR. We conclude from the observation that when we gave initially these antagonists, we could differentiate between effects of the antagonists. In other words, we performed experiments in which we first applied cilostamide and retatrutide and then we added as an antagonist either exendin9-39, ProGIP, or SC203979. Then, we calculate the effect of the antagonist compared to the force elicited by retatrutide before this antagonist.

The fact that cilostamide augmented the efficacy of FOC in HAP supports the view that the positive inotropic effect of retatrutide is mediated via cAMP. The assumption that retatrutide acted via activation of AC may be confirmed by comparing the action of retatrutide with that of isoprenaline. Isoprenaline increased cAMP levels, activated PKA, inhibited PP1, and increased the phosphorylation state of phospholamban (Neumann et al. 1991): moreover, the positive inotropic effect of isoprenaline was reduced by PIA and carbachol which interfered at various steps in this cascade. The positive inotropic effect of isoprenaline is further amplified by trigger calcium cations that pass through the L-type calcium channel (LTCC). The LTCC is inhibited by calcium antagonists like nifedipine. We suggest that in a similar pattern, PIA, carbachol, and nifedipine inhibit at various steps (Fig. 1) the signal transduction of retatrutide. One could readily explain why retatrutide nevertheless to minor extent elevated FOC via GCGR. Indeed, we reported recently that whereas glucagon per se failed to augment FOC in HAP, in the presence of elevated cAMP (cilostamide added), glucagon increased FOC (Neumann et al. 2024a, b). Now one could surmise that in the presence of elevated cAMP, also retatrutide might elevate FOC via CGCR. This elevation in our study may stem from cilostamide that inhibited degradation of cAMP and thus elevated cAMP and protein phosphorylation in HAP (Rayo Abella 2023). Moreover, if we assume that retatrutide excites effectively GLP1-R, this translates into an increase in cellular cAMP. Hence, both cilostamide and the GLP-1R stimulation most likely elevated cAMP in HAP. These two effects combined might tip the scales such that retatrutide can elevate FOC to some extent also via GCGR.

The hypothesis that retatrutide works via cAMP is strengthened by several other arguments. For instance, it is widely taken for granted that the anti-β-adrenergic effects of carbachol and PIA can be derived from an inhibitory action of carbachol and PIA on adenylyl cyclase activity in heart membranes. Hence, if retatrutide acted by stimulation of adenylyl cyclase, then an inhibition of this cyclase by carbachol and PIA would explain the negative inotropic effect of carbachol and PIA in the presence of retatrutide.

**Fig. 7:**
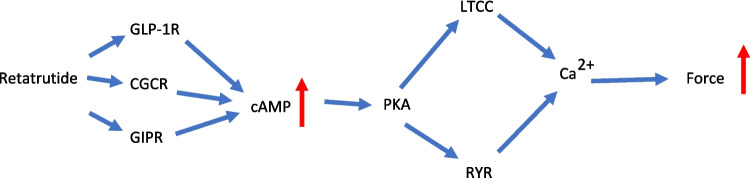
Schematic summary of positive inotropic effect of retatrutide in HAP. Retatrutide stimulates three receptors, namely, the glucagon receptors (GCGR), the glucose-dependent insulinotropic polypeptide (GIP) receptors (GIPR), and the glucagon-like peptide-1 receptors (GLP-1R). This combined stimulate activates adenylyl cyclase to increase cAMP in the human atrial cardiomyocytes. This cAMP activates the cAMP-dependent protein kinase (PKA). This PKA activates by phosphorylation the ryanodine receptor (RyR) and the L-type Ca^2+^ channel (LTCC). The RYR and the LTCC open; together, this leads finally to higher concentrations of Ca^2+^ at the myofibrils. As a consequence, the force of contraction is rising

In this context, our data with nifedipine can be understood. Isoprenaline via cAMP and the cAMP-dependent protein kinase phosphorylates thereby increases the open probability of the L-type calcium channel (Fig. 1). This pathway would be used by retatrutide (Fig. 6). The opening of the LTCC contributes to the positive inotropic effect of isoprenaline, and when this opening is impaired by nifedipine, the positive inotropic effect of isoprenaline can be diminished. The same is in all likelihood taking place with retatrutide: retatrutide leads to the opening of the LTCC, and this opening is impaired by nifedipine (Fig. 7). This plausibly explains why nifedipine reduces the positive inotropic effect of retatrutide in HAP. Looking from a divert angle, one can also argue this fortifies our assumption that the retatrutide exerts its positive inotropic effect via a cAMP involving process.

Others reported before us that exenatide or GLP-1 increased FOC in HAP (Wallner et al. 2015). We also reported that semaglutide and liraglutide (other selective GLP-1 agonists) augmented FOC in HAP (Neumann et al. 2024a, b). These studies concur that GLP-1R stimulation can raise FOC in HAP. Therefore, these data support our hypothesis that retatrutide, an agonist at recombinant GLP1-R in cell culture, acts in part via GLP1-R in HAP.

Retatrutide was studied for the treatment type 2 diabetes and is probably useful to treat obesity. As concerns cardiac effects: they are likely to occur at the therapeutic drug concentrations of retatrutide: under our experimental conditions, retatrutide had a significant positive inotropic effect. It is uncertain whether these findings are detrimental or beneficial for the patient.

We failed to test the effects of retatrutide on the sinus node of man directly. Hence, we cannot prove that retatrutide directly stimulated the sinus node in the human heart but only present evidence in mouse right atrial preparations for a positive chronotropic effect of retatrutide in a companion paper and in abstract form (Neumann et al. 2025). We did not have the opportunity to study contractility in human ventricular tissue for lack of access. Others reported that only about 17% of patients show a positive inotropic effect to GLP-1 stimulation in the ventricle, and no response to glucagon receptor stimulation and no studies on GIP receptors stimulation in the human ventricle are available (Neumann et al. 2025b). In the human ventricle, glucagon is debated to raise FOC. Some papers found effects (Parmley et al. 1970 cf. Neumann et al. 2023b) more recent papers failed to do so (Aranda-Domene et al. 2023). We speculate here that it might be possible for others to detect a positive inotropic effect via GCGR in the isolated human ventricle easily when they amplify cAMP levels by concomitant application of cilostamide and retatrutide.

Limitations of the study: as mentioned above in both monkeys and humans, retatrutide elevated the heart rate and decreased the blood pressure (Coskun et al. 2022, Jastreboff et al. 2023). From our data, it seems clear that retatrutide can act in cardiomyocytes in the HAP. From our data, we cannot conclude that retatrutide acts on sinus node cells in the human heart. Hence, from our data, one cannot decide whether retatrutide increases beating rate in patients in a direct or an indirect way.

In summary, we can now address the hypotheses raised in the Introduction in this way: retatrutide increased the FOC in HAP via GLP-1R, GCGR, and GIPR.

## Data Availability

All source data for this work (or generated in this study) are available upon reasonable request.
